# 2-Allylphenol Reduces IL-1*β* and TNF-*α*, Promoting Antinociception through Adenosinergic, Anti-Inflammatory, and Antioxidant Mechanisms

**DOI:** 10.1155/2019/1346878

**Published:** 2019-03-31

**Authors:** Humberto de Carvalho Aragão Neto, Diogo Vilar da Fonsêca, Renan Marinho Braga, Marcus Tullius Scotti, Terezinha Weyne Araújo Borges do Nascimento, Davidson Barbosa Assis, Sandra Rodrigues-Mascarenhas, Luiz Henrique Agra Cavalcante Silva, José Guilherme Ferreira Marques Galvão, Hugo Alexandre Oliveira Rocha, Arthur Antunes Jacome Vidal, José Maria Barbosa Filho, Reinaldo Nóbrega de Almeida

**Affiliations:** ^1^Institute of Drugs and Medicines Research, Federal University of Paraíba, João Pessoa 58051-085, Brazil; ^2^Department of Biochemistry, Federal University of Rio Grande do Norte, Natal 59072-970, Brazil

## Abstract

2-Allylphenol (2-AP) is a synthetic phenylpropanoid, structurally related to cardanol, thymol, and ortho-eugenol. Phenylpropanoids are described in the literature as being capable of promoting biological activity. Due to the similarity between 2-AP and other bioactive phenylpropanoids, the present research aims at evaluating the antioxidant, antinociceptive, and anti-inflammatory potential of 2-AP in silico, *in vitro*, and *in vivo*. At 30 min prior to the start of *in vivo* pharmacological testing, administration of 2-AP (25, 50, 75, and 100 mg/kg i.p.), morphine (6 mg/kg i.p.), dexamethasone (2 mg/kg s.c.), or vehicle alone was performed. In the acetic acid-induced abdominal writhing tests, pretreatment with 2-AP significantly reduced the number of abdominal writhes, as well as decreased licking times in the glutamate and formalin tests. Investigation of the mechanism of action using the formalin model led to the conclusion that the opioid system does not participate in its activity. However, the adenosinergic system is involved. In the peritonitis tests, 2-AP inhibited leukocyte migration and reduced releases of proinflammatory mediators TNF-*α* and IL-1*β*. *In vitro* antioxidant assays demonstrated that 2-AP presents significant ability to sequester superoxide radicals. *In silico* docking studies confirmed interaction between 2-AP and the adenosine A2a receptor through hydrogen bonds with the critical asparagine 253 residues present in the active site. Investigation of 2-AP demonstrated its nociception inhibition and ability to reduce reactive oxygen species. Its interaction with A2a receptors may well be related to proinflammatory cytokines TNF-*α* and IL-1*β* reduction activity, corroborating its antinociceptive effect.

## 1. Introduction

Nociception is the transmission of signals captured by free nerve endings in response to noxious stimuli; this stimulus is converted into electrical signals that reach the spinal cord, thalamus, and cerebral cortex [1]. Increased oxidative stress and inflammation are factors capable of amplifying pain. The exacerbated production of inflammatory mediators, such as cytokines (TNF-*α* and IL-1*β*), sensitizes and/or activates nociceptive neurons. This sensitization initially occurs in nociceptive neurons near injured tissue, leading to increases in pain sensation at the site [2, 3]. Inflammatory mediators induce recruitment of neutrophils responsible for producing reactive oxygen species (ROS), such as the superoxide anion and its derivatives. Cytokines and ROS released by macrophages and neutrophils activate and sensitize nociceptive neurons, resulting in more intense pain [4].

To promote patient comfort, the sensation of pain should be reduced, and although there are several drugs for this purpose, research continues for new compounds that are more potent, safer, and have fewer side effects. Within this context, medicinal plants have been gaining popularity for treating disease. *Ginkgo biloba* (GB) extract is one of the most commercialized products in the world, whose pharmacological potential is ample [5]. Studies have shown its anti-inflammatory activity in paw edema models, as well as its antinociceptive effect in thermal and chemical models of pain induction [6]. Zayed et al. [7] found that treatment with GB extract is able to protect the kidneys of mice presenting type 2 diabetes from nephrotoxic damage, through reducing oxidative stress. It is shown that GB extract also exerts protective effects in the brain and has been able to reverse *β*-peptide-induced and hydrogen peroxide-stimulated isoprostane production [8]. Its essential oil (EO) presents antioxidant efficacy in both *in vitro* and *in vivo* studies [9]. Previous research, involving GB essential oil, identified 68 compounds; one was cardanol, a bioactive compound already described in the literature for having antimicrobial, antioxidant, antitumor, and antifungal activity and improving cognitive ability and learning [10–12].

2-Allylphenol (2-AP) is a synthetic phenylpropanoid, structurally related to cardanol, thymol, and ortho-eugenol. It is a widely marketed fungicide in China under the name Yinguo, where studies attribute its activity by inducing cyanide-resistant respiration, causing an ATP decrease, and inhibiting the fungal pathogen respiration [13]. However, 2-AP has no other properties described in the literature, making this study relevant to investigate its capacity to reduce pain, inflammation, and oxidative stress.

Due to the similarity between 2-AP and other bioactive phenylpropanoids, the present research aims at evaluating the antioxidant potential of 2-AP, which was determined *in vitro* through total antioxidant capacity, DPPH (1,1-diphenyl-2-picrylhydrazyl) sequestering activity, hydroxyl radical sequestering activity, and superoxide sequestering activity tests; at elucidating its antinociceptive activity, which was performed *in vivo* experimental models of acetic acid induced abdominal contortions, glutamate test, and formalin-induced paw-licking test; at investigating its possible mechanism of action, which was made in silico methodology of docking and confirmed *in vivo* with the aforementioned formalin test, to evaluate the opioid and adenosinergic systems involvement in 2-AP antinociceptive effect; and its anti-inflammatory activity were analyzed by using *in vivo* model of carrageenan-induced peritonitis and *in vitro* assay of peritoneal fluid dosage of TNF-*α* and IL-1*β*.

## 2. Material and Methods

### 2.1. Animals

Three-month-old male Swiss mice (25-35 g) from the Prof. Dr. Thomas George Bioterium, at the Federal University of Paraíba, were kept under controlled temperature conditions (21 ± 1°C), with free access to water and feed and a light/dark cycle of 12 hours. All the experimental procedures were previously approved by the CEPA—Committee of Ethics in Animal Research at UFPB, under certificate # 0201/2013.

### 2.2. Drugs

2-AP, glutamate, and MK-801 were purchased from Sigma (St. Louis, MO, USA). Formaldehyde 37% (Vetec, Brazil), morphine hydrochloride (Vetec, Brazil), caffeine (Merck, USA), and naloxone hydrochloride (Cristália, Brazil) were also purchased and used in this study. All drugs were diluted in distilled water except 2-AP which required Tween 80 and distilled water. The other solvents and chemicals used in this study were purchased from Sigma-Aldrich® (São Paulo, São Paulo, Brazil).

### 2.3. Test of Acetic Acid-Induced Abdominal Contortions

The mice were divided into five groups (*n* = 8) and received administrations of 5% Tween 80, morphine (6 mg/kg, i.p.), and 2-AP (25, 50, 75, and 100 mg/kg, i.p.) 30 min before intraperitoneal injections of 1% acetic acid. After this nociceptive stimulus administration, the animals were placed individually in observation boxes where they remained for 20 min, counting the total number of abdominal contortions during the final 15 min.

### 2.4. Glutamate Testing

The animals were treated with vehicle, 2-AP (50, 75, and 100 mg/kg, i.p.), or MK-801 (0.03 mg/kg, i.p. NMDA antagonist) 30 min before intraplantar injection of glutamate (30 *μ*mol/20 *μ*L). The mice were observed for 15 min after administration of the pain stimulus, and the paw licking time was measured as indicative of nociception.

### 2.5. Formalin Test

After vehicle treatments of 2-AP (50, 75, and 100 mg/kg, i.p.) or morphine (6 mg/kg, i.p.), the mice received 20 *μ*L of 1% formalin diluted in distilled water in the subplantar region. The animals were immediately placed in a glass box to record licking times. The nociceptive behavior was recorded in two phases after administration of formalin: phase 1 (0-5 min) and phase 2 (15-30 min), respectively, representing neurogenic and inflammatory pain responses.

### 2.6. Opioid System Involvement in 2-AP Antinociceptive Effect

In order to evaluate the participation of the opioid system in the 2-AP antinociceptive effect, different groups of animals (*n* = 8) were treated with vehicle, 2-AP (100 mg/kg i.p.), morphine (6 mg/kg i.p.), or naloxone, a nonselective opioid antagonist (5 mg/kg, s.c.) [14]. Two groups received pretreatment with naloxone (5 mg/kg, s.c.), at 15 min prior to administration of 2-AP (100 mg/kg i.p.) or morphine (6 mg/kg i.p.). After a period of 30 min, the effects of these treatments in the formalin test were evaluated.

### 2.7. Adenosinergic System Involvement in the Antinociceptive Effect of 2-AP

Potential participation of the adenosinergic system in the mechanism of action of 2-AP was studied using caffeine as a nonselective adenosine receptor antagonist [15]. The animals were divided into different groups (*n* = 8) where one group received caffeine (10 mg/kg, s.c.) 15 min prior to administration of 2-AP (100 mg/kg, i.p.). The other groups received only vehicle, caffeine, or 2-AP. After 30 min, the mice were submitted to the formalin test.

### 2.8. Carrageenan-Induced Peritonitis Test

Leukocyte migration was induced by intraperitoneal administration of carrageenan (1%, 300 *μ*L) in mice (*n* = 8) treated 30 min earlier with vehicle, 2-AP (100 mg/kg, i.p.), or dexamethasone (2 mg/kg, s.c.) Four hours after administration, the mice were euthanized, and the peritoneal cavity was filled with 2 mL of saline. From the peritoneal fluid, an aliquot was removed to determine total leukocyte count. The results were expressed as leukocytes/mL.

### 2.9. Peritoneal Fluid Dosing of TNF-*α* and IL-1*β*

Four hours after administration of carrageenan to the groups pretreated with vehicle, 2-AP (100 mg/kg, i.p.), or dexamethasone (2 mg/kg, s.c.), the peritoneal fluid was removed and centrifuged at 16,100 × g/5 min/4°C. The supernatant was removed for TNF-*α* and IL-1*β* measurements using ELISA sandwich technique and performed according to the manufacturer's instructions (eBioscience, San Diego, USA). The cytokine quantities were calculated from standard curves and expressed in total per milliliter (pg/mL).

### 2.10. DPPH (1,1-Diphenyl-2-picrylhydrazyl) Free Radical Scavenging Activity

DPPH radical scavenging activity was measured using the Shimada et al. [16] method with some modification. We added 0.1 mL of different 2-AP concentrations (0.25, 0.5, 1, 2, and 5 mg/mL), and finally 1 mL of a 0.1 mM DPPH/methanol (Fluka) solution. The mixture was then stirred vigorously for 30 min at room temperature, and the absorption of the resulting solution was measured at 517 nm. The percentage inhibition of the DPPH radical was calculated by comparing the test results with those of the control (not treated with 2-AP) and the following equation: scavenging activity (%) = (1 − *A*1/*A*0) × 100, where *A*0 is the control absorbance and *A*1 is sample absorbances.

### 2.11. Evaluation of Hydroxyl Radical (OH^−^) Removal Activity

2-AP hydroxyl radical-scavenging activity was evaluated using Fenton's reaction (Fe^2+^ + H_2_O_2_ → Fe^3+^ + OH^−^ + OH^.^). Different 2-AP concentrations (0.25, 0.5, 1.0, 1.5, and 2 mg/mL) were added to a reaction mixture containing 150 mM of sodium phosphate buffer (pH 7.4), 8 mM sodium salicylate, 40 mM iron II sulfate, and 40 mM EDTA. The reaction mixture was treated with hydrogen peroxide (10% *v*/*v*), then stirred and incubated in a water bath (37°C) for 1 hour. The absorbance was measured (510 nm) with a spectrophotometer. Control (mixture without samples) and blank mixtures were also prepared. Gallic acid (0.5 mg/mL) was also used. Hydrogen peroxide was not added to the blank tubes. The results were expressed as the percentage of hydroxyl radical-scavenging activity, as shown in equation 1.

(1) $$\begin{array}{c} \%\text{radical scavenging}=\left(\left[{\text{A}}_{\text{control}}-{\text{A}}_{\text{sample}}\right]/\left[{\text{A}}_{\text{control}}-{\text{A}}_{\text{blank}}\right]\right)\times 100, \end{array}$$

where A_control_ is the control tube absorbance, A_sample_ is the sample tube absorbance, and A_blank_ is the blank tube absorbance.

### 2.12. Superoxide Anion (O_2_^−^) Scavenging Activity

This assay was based on the capacity of 2-AP (0.1, 0.25, and 0.5 mg/mL) to inhibit photochemical reduction of nitro-blue tetrazolium (NBT) in a riboflavin-light-NBT system [17]. Each 3 mL reaction mixture contained 50 mM phosphate buffer (pH 7.8), 13 mM methionine, 2 *μ*M riboflavin, 100 *μ*M EDTA, NBT (75 *μ*M), and 1 mL of sample solution. After blue formazan production, the increase in absorbance at 560 nm after 10 min of exposure to fluorescent light was determined. Identical tubes with the reaction mixture were kept in the dark and were used as blanks. Gallic acid (0.5 mg/mL) was used for positive control. The results were expressed as the percentage of superoxide radical-scavenging activity, as shown in equation 2.

(2) $$\begin{array}{c} \%\text{radical scavenging}=\left(\left[{\text{A}}_{\text{control}}-{\text{A}}_{\text{sample}}\right]/\left[{\text{A}}_{\text{control}}-{\text{A}}_{\text{blank}}\right]\right)\times 100, \end{array}$$

where A_control_ is the control tube absorbance, A_sample_ is the sample tube absorbance, and A_blank_ is the blank tube absorbance.

### 2.13. Total Antioxidant Capacity (TAC)

This assay was based on reduction of Mo (VI) to Mo (V) by 2-AP and subsequent formation of a green phosphate/Mo (V) complex at acid pH [18]. Tubes containing 2-AP and a reagent solution (0.6 M sulfuric acid, 28 mM sodium phosphate, and 4 mM ammonium molybdate) were incubated at 95°C for 90 min. After the mixture had cooled to room temperature, the absorbance of each solution was measured at 695 nm against a blank. The antioxidant capacity was expressed as mg ascorbic acid/g, described as ascorbic acid equivalent (AAE/g).

### 2.14. Docking Studies

The structure of 2-AP was used as input data for Marvin 14.9.1.0, 2014, ChemAxon (http://www.chemaxon.com). We used Standardizer Software [JChem 14.9.1.0, 2014; ChemAxon (http://www.chemaxon.com)] to canonize the structure, add hydrogens, perform aromatic form conversions, clean the molecular graph in three dimensions, and save the compounds in sdf format [19].

The human adenosine A2a receptor structures in complex with endogen ligand (PDB ID 2YDO) and agonist UKA (PDB ID 3QAK) were downloaded from the Protein Data Bank (http://www.rcsb.org/pdb/home/home.do) [20, 21]. The 2-AP structure was submitted to molecular docking using the Molegro Virtual Docker, v. 6.0.1 (MVD) [22]. All of the water compounds were deleted from the enzyme structure, and the enzyme and compound structures were prepared using the same default parameter settings from the same software package (score function: MolDock score; ligand evaluation: internal ES, internal HBond, Sp2–Sp2 torsions, all checked; number of runs: 10 runs; algorithm: MolDock SE; maximum interactions: 1500; max. population size: 50; max. steps: 300; neighbor distance factor: 1.00; max. number of poses returned: 5). The docking procedure was performed using a GRID of 15A° in radius and 0.30 in resolution to cover the ligand-binding site for the adenosine A2a receptor structure. The Moldock score [GRID] algorithm was used as the score function, and the Moldock search algorithm was used [22].

### 2.15. Molecular Surface Interactions

Three-dimensional structures (3D) were used as input data in the Volsurf+ program v. 1.0.7 and were subjected to molecular interaction fields (MIF) [23] to generate molecular surfaces using the following probes: N1 (amide nitrogen-hydrogen bond donor probe), O (carbonyl oxygen-hydrogen bond acceptor probe), OH2 (water probe), and DRY (hydrophobic probe).

### 2.16. Statistical Analysis

The results were analyzed using one-way analysis of variance (ANOVA) method followed by the Dunnett test for comparison between the means. Data were expressed as mean±SEM (standard error of the mean), and values were considered significant when they presented a level of significance (*p*) of less than 0.05.

## 3. Results

### 3.1. Effect of 2-AP in the Acetic Acid-Induced Writhing Test

**Figure 1:**
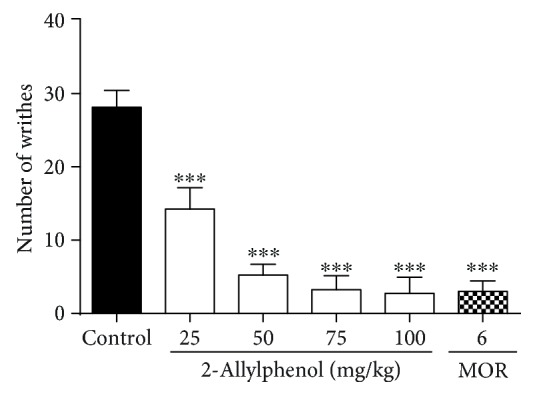
Effect of 2-AP on the number of contortions induced by acetic acid. Mice were pretreated with 2-AP (25, 50, 75, and 100 mg/kg, i.p.), morphine (MOR: 6 mg/kg, i.p.), and vehicle 30 min before administration of acetic acid (1% i.p.). Each column represents the mean±S.E.M. (*n* = 8). Statistical analysis: one-way ANOVA followed by the Dunnett test. ^∗∗∗^ *p* < 0.001; (2-AP)=*vs.* control and (MOR)=*vs.* control.

2-AP at doses of 25 (14.3 ± 2.9), 50 (5.3 ± 1.5), 75 (3.3 ± 1.9), and 100 (2.8 ± 2.2) mg/kg presented a significant reduction in the number of contortions induced by acetic acid as compared to the control group (28.5 ± 2.3) (*p* < 0.001), with respective inhibition percentages of 49.4%, 80.1%, 88.3%, and 90.0%. Morphine as expected reduced the contortion response by 89.3% (3.0 ± 1.4; *p* < 0.001) (Figure 1).

### 3.2. Effect of 2-AP in the Glutamate Test

**Figure 2:**
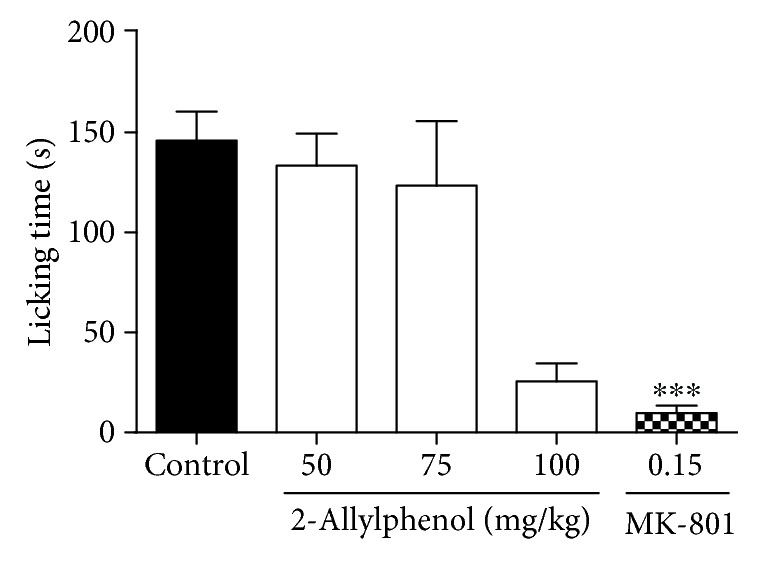
Effect of 2-AP on glutamate-induced licking behavior. Mice were pretreated with vehicle, 2-AP (50, 75, and 100 mg/kg, i.p.), and MK-801 (0.15 mg/kg, i.p.) 30 min before administration of glutamate (30 mmol/paw). Each column represents the mean±S.E.M. (*n* = 8). Statistical analysis: one-way ANOVA followed by the Dunnett test. ^∗∗∗^ *p* < 0.001; (2-AP)=*vs.* control and (MK-801)=*vs.* control.

Pretreatment with 2-AP at a dose of 100 (25.5 ± 9.1 s) mg/kg reduced the glutamate-induced licking time, with an inhibition value of 82.5%, compared to the control group (*p* < 0.001). The two lower doses of 2-AP (50 and 75 mg/kg) were not effective in the glutamate test. MK-801, an NMDA receptor antagonist, reduced glutamate-induced nociceptive behavior by 93.3% (9.8 ± 3.6 s; *p* < 0.001) (Figure 2).

### 3.3. Effect of 2-AP in the Formalin Test

**Figure 3:**
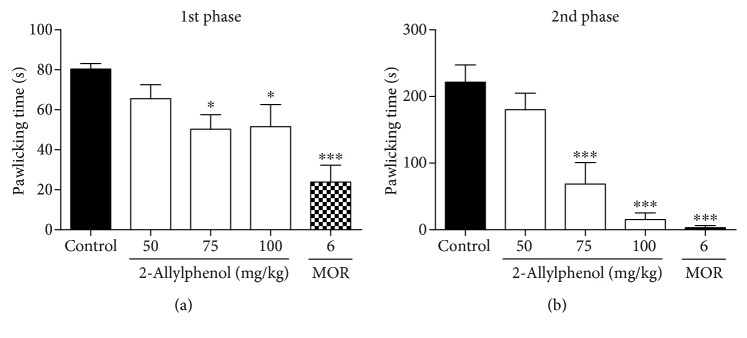
Effect of 2-AP on the paw licking time. Mice were pretreated with vehicle, 2-AP (50, 75, and 100 mg/kg, i.p.), and morphine (MOR: 6 mg/kg, i.p.) 30 min before subplantar formalin administration (1%, 20 *μ*L/paw). Each column represents the mean±S.E.M. (*n* = 8). Statistical analysis: one-way ANOVA followed by the Dunnett test. ^∗∗∗^ *p* < 0.001; (2-AP)=*vs.* control and (MOR)=*vs.* control.

2-AP at doses of 75 and 100 mg/kg demonstrated a significant reduction in licking time in both phases of the formalin test when compared to the control group (80.4 ± 2.7 s in the first phase and 221.3 ± 26.1 s in the second phase) (*p* < 0.05), with respective inhibition rates of 37.4% (50.3 ± 7.3 s) and 35.8% (51.6 ± 11.1 s) in the first phase and 69.0% (68.7 ± 32.2 s) and 93.0% (15.4 ± 10.0 s) in the second phase. Morphine reduced the behavior in both phases, respectively, 70.2% (23.9 ± 8.4 s) and 98.6% (3.2 ± 3.2 s) (*p* < 0.001) (Figure 3).

### 3.4. Opioid System Involvement in 2-AP Antinociceptive Effects

**Figure 4:**
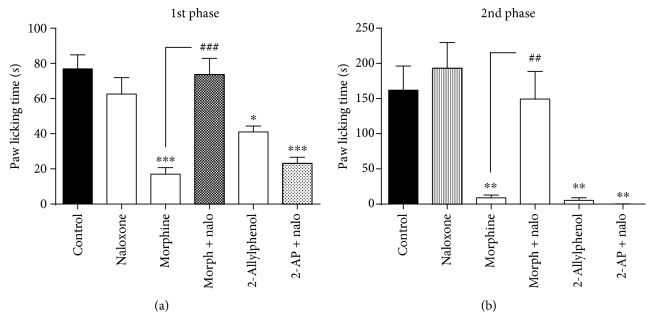
Effect of pretreatment with naloxone on antinociception caused by 2-AP in the formalin test. Mice were pre-treated with naloxone (5 mg/kg, s.c.) at 15 min before treatment with 2-AP (100 mg/kg, i.p.) or morphine (6 mg/kg, i.p.). 30 min after the treatment they received a subplantar administration of formalin (1%, 20 μL/paw). Each column represents the mean±S.E.M. (*n* = 8). Statistical analysis: one-way ANOVA followed by the Dunnett test. ^∗^ *p* < 0.05; ^∗∗^ *p* < 0.01; ^∗∗∗^ *p* < 0.001; (2-AP)=*vs.* control, (morphine)=*vs.* control, (naloxone)=*vs.* control (morphine+nalo)=*vs.* control and (2-AP+nalo)=*vs.* control. ^##^*p* < 0.01; ^###^*p* < 0.001; (morph+nalo)=*vs.* morphine.

The antinociceptive effect of 2-AP (100 mg/kg, i.p.) was not reversed as a function of prior naloxone administration (*p* < 0.001). The effect produced by morphine was significantly reversed (Figure 4).

### 3.5. Adenosinergic System Involvement in the Antinociceptive Effect of 2-AP

**Figure 5:**
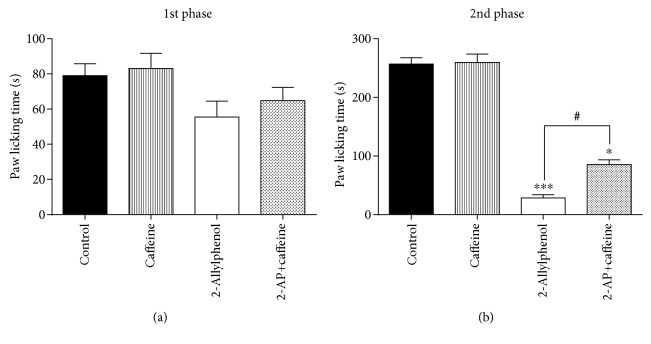
Effect of caffeine pretreatment on antinociception caused by 2-AP in the formalin test. Mice were treated with caffeine (10 mg/kg, s.c.), at 15 min before treatment with 2-AP (100 mg/kg, i.p.). The other groups were treated with vehicle, caffeine, or 2-AP 30 min before subplantar administration of formalin (1%, 20 *μ*L/paw). Each column represents the mean±S.E.M. (*n* = 8). Statistical analysis: one-way ANOVA followed by the Dunnett test. ^∗^ *p* < 0.05; ^∗∗∗^ *p* < 0.001; (2-AP)=*vs.* control, (caffeine)=*vs.* control and (2-AP+caffeine)=*vs.* control. ^#^*p* < 0.05; (2-AP)=*vs.* 2-AP+caffeine.

Previous administration of caffeine, an adenosine receptor antagonist, was able to reverse the antinociceptive effect of 2-AP (100 mg/kg, i.p.) expressively in the second phase of the formalin test (*p* < 0.05) (Figure 5).

### 3.6. Effect of 2-AP in the Carrageenan-Induced Peritonitis Test

**Figure 6:**
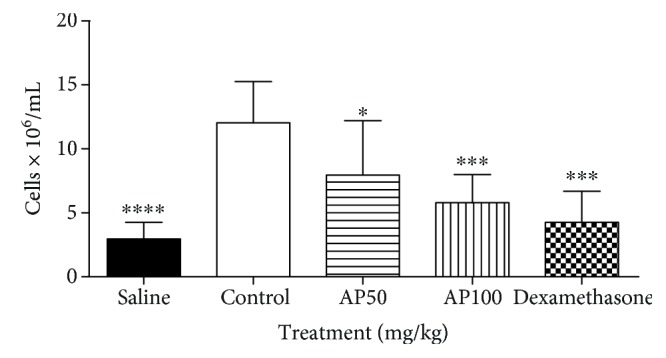
Effect of 2-AP administration on total leukocyte migration in the carrageenan-induced peritonitis test. Injection of carrageenan (1% 300 *μ*L) into the peritoneal cavity of the mice, 30 min after vehicle administration, 2-AP (50 and 100 mg/kg, i.p.), and dexamethasone (2 mg/kg s.c.) were administered. Each column represents the mean±S.E.M. (*n* = 8). Statistical analysis: one-way ANOVA followed by the Dunnett test. ^∗^ *p* < 0.05; ^∗∗∗^ *p* < 0.001; ^∗∗∗∗^ *p* < 0.0001; (AP)=*vs.* control, (dexamethasone)=*vs.* control and (saline)=*vs.* control.

2-AP administered 30 min before administration of carrageenan (1% 300 *μ*L) inhibited leukocyte migration at doses of 50 and 100 mg/kg by 35.9% (7.9 ± 1.6 × 10^6^ leukocytes/mL) and 51.75% (5.8 ± 0.8 × 10^6^ leukocytes/mL), respectively, when compared to the controls (12.0 ± 1.1 × 10^6^ leukocytes/mL) (*p* < 0.001). As expected, dexamethasone reduced leukocyte migration by 64.6% (4.3 ± 1.4 × 10^6^ leukocytes/mL) (*p* < 0.001) (Figure 6).

### 3.7. Effect of 2-AP on TNF-*α* and IL-1*β*

**Figure 7:**
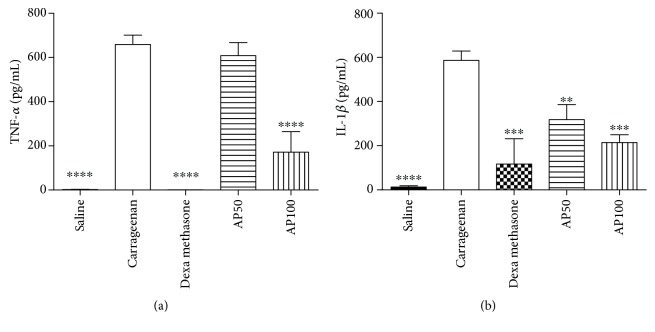
Effect of 2-AP administration in the peritoneal cavity 4 h after intraperitoneal administration of carrageenan on levels of TNF-*α* and IL-1*β*. Mice were pretreated with vehicle, 2-AP (50 and 100 mg/kg, i.p.), and dexamethasone (2 mg/kg s.c.) 30 min prior to carrageenan injection. Each column represents the mean±S.E.M. (*n* = 8). Statistical analysis: one-way ANOVA followed by the Dunnett test. ^∗∗^ *p* < 0.01; ^∗∗∗^ *p* < 0.001; ^∗∗∗∗^ *p* < 0.0001; (saline)=*vs.* carrageenan, (dexamethasone)=*vs.* carrageenan and (AP)=*vs.* carrageenan.

Four hours after administration of carrageenan, 2-AP at 100 mg/kg reduced TNF-*α* levels by 74.0% (172.2 ± 93.2) compared to the controls (662.5 ± 42.5) (*p* < 0.0001). To assess IL-1*β* levels, 2-AP at doses of 50 and 100 mg/kg reduced IL-1*β* by 45.8% (319.5 ± 69.1) and 63.4% (215.6 ± 35.4), respectively, when compared to the control group (589.4 ± 42.2) (*p* < 0.0001). Dexamethasone reduced levels of TNF-*α* by 99.8% (0.7 ± 0.8) (*p* < 0.0001) and IL-1*β* by 79.9% (117.9 ± 115.0) (*p* < 0.001) (Figure 7).

### 3.8. Effect of 2-AP in DPPH Radical Sequestering Activity

The present study demonstrated DPPH radical sequestering activity by 2-allylphenol in differing concentrations (0.25, 0.5, 1.0, 2.0, and 5.0 mg/mL). At the concentration of 0.25 mg/mL, sequestering activity was 34.3%, greater than the activity occurring at the larger concentrations which did not present significant differences between them (Table 1).

### 3.9. Effect of 2-AP in Hydroxyl Radical Sequestering Activity

In the hydroxyl radical sequestering activity tests, for 2-allylphenol at the concentration of 0.5 mg/mL, there was no inhibition of the hydroxyl radical; however, in the other concentrations (1.0, 1.5, and 2.0 mg/mL), there were activity increases, with significant differences between the concentrations tested (Table 2).

### 3.10. Effect of 2-AP in Superoxide Radical Sequestering Activity

2-Allylphenol presented high percentages of superoxide radical sequestration at concentrations of 0.1 (88.40 ± 4.2%), 0.25 (96.73 ± 0.19%), and 0.5 mg/mL (96.08 ± 1.11%), with no significant differences between them (Table 3).

### 3.11. Effect of 2-AP in Total Antioxidant Capacity (TAC)

The total antioxidant capacity was expressed in gallic acid equivalents (mg gallic acid/g sample). 2-AP presented a TAC of 319.97 ± 0.38 mg/g.

### 3.12. Analysis of 2-AP in Docking Studies

**Figure 8:**
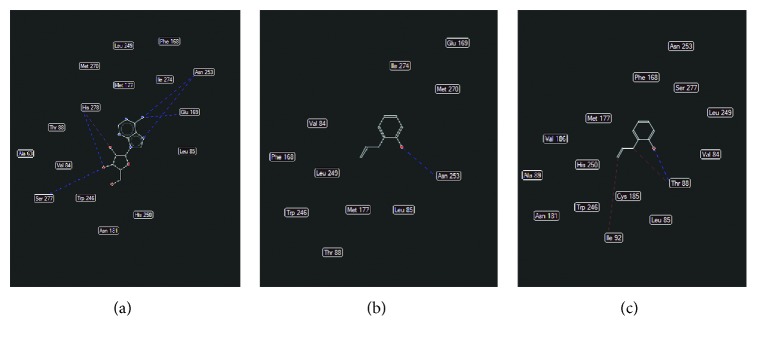
Hydrogen-bonding interactions for (a) adenosine, (b) 2-AP, and (c) 2-AP (without template) (PDB ID 2YDO). Blue dash lines represent H-bond interactions; brown dash lines represent steric clashes.

**Figure 9:**
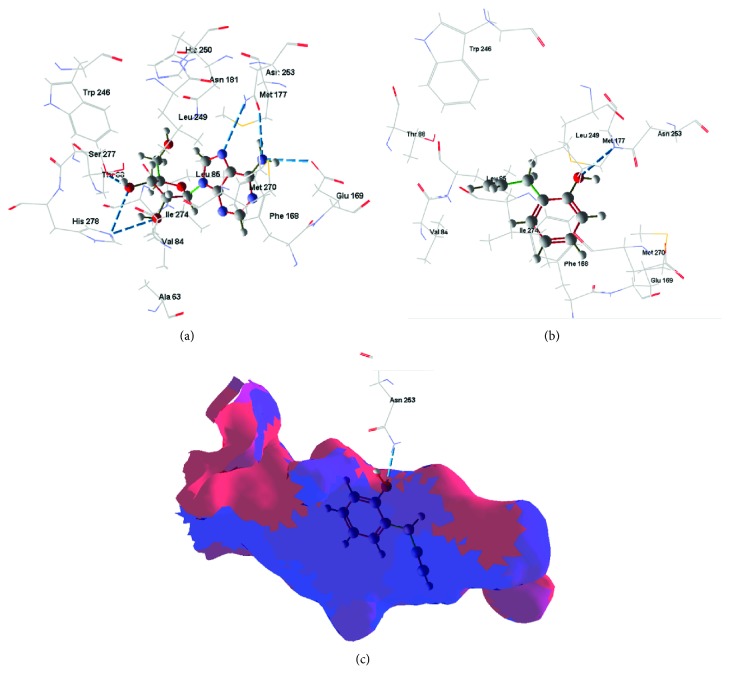
Best docking results for adenosine (a) and 2-AP (b) in the A2a active site. Blue dash lines represent H-bond interactions; brown dashed lines represent steric clashes. (c) Binding conformations for 2-AP at the adenosine A2a receptor (PDB ID 2YDO) active site. The blue surface represents favorable hydrophobic areas; the red surface represents favorable hydrophilic areas.

**Figure 10:**
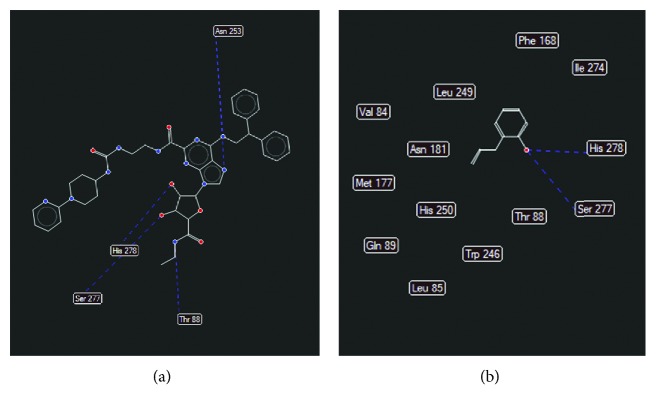
Hydrogen bonding interactions for (a) UKA and (b) 2-AP (PDB ID 3QAK). Blue dash lines represent H-bond interactions; brown dash lines represent steric clashes.

**Figure 11:**
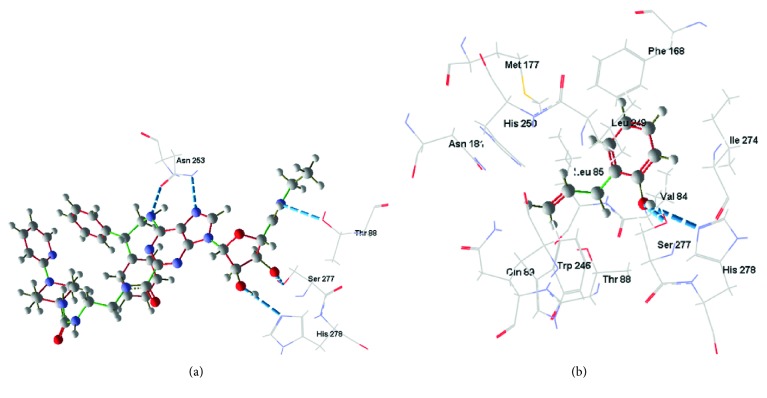
Best docking results for UKA (a) and 2-AP (b) in the A2a (PDB ID 3QAK) active site. Blue dash lines represent H-bond interactions; brown dash lines represent steric clashes.

**Figure 12:**
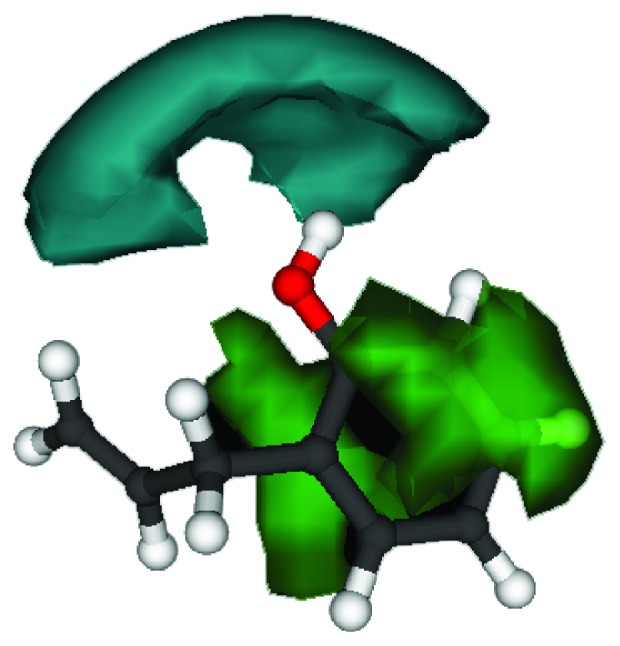
Grid 3D molecular fields of 2-AP with DRY probe. The green contours are hydrophobic regions shown at -1.0 kcal mol^−1^; cyan contours are hydrophilic regions at -2.0 kcal mol^−1^.

In 2YDO crystallography, 2-AP presented interactions with critical residues of threonine (Thr) 88 and asparagine (Asn) 253 (Figures 8 and 9) forming hydrogen bonds. In 3QAK crystallography, 2-AP interacted with critical residues of histidine (His) 278 and serine (Ser) 277 (Figures 10 and 11), also forming hydrogen bonds. Adenosine energy values are as follows: MolDock -104.13 kJ/mol and rerank -90.46 (2YDO); 2-AP: MolDock -59.18 kJ/mol and rerank -52.83 (2YDO); UKA: MolDock -230.52 kJ/mol and rerank -148.70 (3QAK); 2-AP: MolDock -52.25 kJ/mol and rerank -47.47 (3QAK). The logP calculated was 2.77 (Figure 12).

## 4. Discussion

The analgesic activity study was initiated with acetic acid-induced contortion testing, a classic model of pain used for screening analgesic and anti-inflammatory substances [24]. Administration of acetic acid to the peritoneal cavity promotes nonselective activation of cationic channels and release of inflammatory mediators, causing nociception characterized by paw extensions and abdomen constriction [25]. The nociceptive effect can be prevented by steroidal and nonsteroidal anti-inflammatories, as well as by centrally acting analgesics [26]. Treatment with 2-AP reduced in a dose-dependent manner the number of abdominal writhes induced by acetic acid, suggesting that the substance may reduce the release of inflammatory mediators or directly inhibit nociception [27]. The data are similar to the results observed by Fonsêca et al. [1], where ortho-eugenol, a molecule analogous to 2-AP, also demonstrated reductions in the number of abdominal writhes as induced by stimulation with acetic acid.

Glutamate is the principal neurotransmitter of the central nervous system, involved in various physiological processes such as memory, learning, and excitotoxicity [28]. Glutamate is involved in the maintenance and onset of pain through the activation of sensory C fibers which transmit the nociceptive impulse to the spinal cord [29]. It is suggested that at least in part, 2-AP performs its antinociceptive activity due to interaction with the glutamatergic system. Accordingly, Li et al. [30] demonstrated that *Ginkgo biloba* extract reduces the influx of calcium in hippocampal neurons, preventing the onset of noxious stimulus.

As a model of neurogenic and inflammatory pain, the formalin test was performed to evaluate the antinociceptive properties of 2-AP. The neurogenic phase, the first phase, is directly related to chemical stimulation of receptors with probable involvement of substance P, glutamate, and bradykinin. The second phase corresponds to inflammatory pain caused by the release of serotonin, histamine, and prostaglandins [31]. Central analgesic drugs inhibit both phases of the formalin test, especially the second phase [32]. Peripherally acting drugs such as nonsteroidal anti-inflammatory drugs inhibit only the second (late) phase [33, 34]. Acute treatment with 2-AP reduced nociceptive behavior in both phases of the formalin test; however, second phase pain reduction was more effective, indicating that the test substance is more potent in its anti-inflammatory mechanisms. The result obtained differs from most phenylpropanoids studied, such as eugenol, methyleugenol, 1-nitro-2-phenylethane, and anethole, which inhibited only the second phase of the formalin test, and thus presenting only anti-inflammatory activities.

Investigation of mechanisms of action is based on the use of known pharmacological antagonists, which, if previously administered, block effects resulting from specific receptor activation and prevent the substance under study from binding to its receptor and promote its activity.

The first system to be investigated was the opioid system. Opioid receptors are expressed in nerves involved in pain transmission (sensory ascending trajectory) and modulation (descending inhibitory trajectory) in sites located at the spinal and supraspinal levels [35].

In order to verify participation of the opioid system in the antinociceptive effect of 2-AP, the animals were previously treated with naloxone (an anatomopathic opioid). The results presented no reversal of 2-AP antinociceptive effect and indicated that such effect is not due to opioid receptor activation. This preliminary result demonstrates a certain advantage over morphine because 2-AP is able to exert similar antinociceptive activity, yet for not acting on the opioid system, it is possible that 2-AP would not cause respiratory depression or other side effects characteristic of opioids.

Participation of the adenosinergic system in the 2-AP mechanism of action was then investigated. Adenosine acts as an endogenous signaling agent, acting on four distinct extracellular G protein-coupled receptors (A1, A2A, A2B, and A3) [36]. Preclinical studies report a diversity of pain models which exhibit antinociceptive properties for A1 receptor agonists, including studies using the formalin-induced paw-lick model [37, 38]. The mechanisms involved in the antinociceptive action of A1 receptors in peripheral regions involve inhibition of cyclic AMP/PKA. In spinal regions, presynaptic inhibition of Ca^2+^ channels followed by postsynaptic hyperpolarization occurs. In supraspinal regions, the mechanism involves interaction with K^+^ channels, increasing their activity and consequent hyperpolarization [39–41]. A2A receptors are expressed peripherally in immune and inflammatory cells and mediate anti-inflammatory activities [42]. A3 receptors are expressed in various organs and in peripheral tissues; including cells that participate in inflammatory responses [43].

In interaction with caffeine, 2-AP presented a reversal of its antinociceptive effects in both phases of the formalin test. From these results, it is suggested that A1, A2A, and/or A3 receptors may be involved in the mechanisms of central and peripheral antinociceptive activity exerted by 2-AP.

In the docking study using crystallography of the A2a adenosine receptor, hydrogen-binding (H-bond) interactions between 2-AP and the critical Asn 253 residue were observed. From the crystallographic structure of adenosine (2YDO), the ligand is capable of forming two hydrogen bonds with the Asn 253 residue, suggesting the relevance of this receptor interaction. The synthetic agonist (UKA), which was designed specifically for high A2a receptor selectivity [21], interacts through residues Asn 253, Thr 88, His 278, and Ser 277. When compared to UKA, we found in 2YDO crystallography that 2-AP exhibits interactions with residues of Asn 253 and Thr 88, and using 3QAK crystallography, we found interactions with His 278 and Ser 277 residues.

In terms of polarity (LogP 2.77), 2-AP presents an intermediate character; its lipophilic/hydrophilic balance can be associated with its residue interactions since Asn, Thr, and Ser are polar group holders and form hydrogens with the hydrophilic region of the molecular surface, represented by the hydroxyl group; whereas the conformation of 2-AP within the active site indicates that its hydrophobic region is accommodated within the protein.

The A2A receptor is peripherally expressed in cells of the immune system and plays a key role in the mechanism of inflammation; its activation is commonly associated with reduced inflammatory status [44]. Antonioli et al. [42] show that its anti-inflammatory mechanism involves decreases in multiple proinflammatory mediators of immune cells. Such receptors are also expressed in pre- and postsynaptic neurons and glial cells, being relevant locations for painful stimuli [45]. Loram et al. [46] in multiple experimental models observed attenuation of neuropathic pain using a selective agonist of the A2A receptor, indicating that its activation causes reductions in microglial and astrocyte production of TNF*α*. Corroborating these findings, Ravani et al. [47] consider A2A agonists as potential alternatives for treatment of both pain and inflammation in patients with rheumatoid arthritis.

The docking studies confirmed an affinity between 2-AP and the A2a receptor; this interaction may be related to a reduction in proinflammatory cytokines TNF-*α* and IL-1*β*, corroborating its antinociceptive effect.

Administration of carrageenan i.p. generates an inflammatory process characterized by leukocyte aggregation and fluid extravasation; with participation of cytokines, nitric oxide, leukotrienes, and PGE2 [44]. Our data suggest that 2-AP reduces carrageenan-induced leukocyte migration as well as eugenol and estragole, both phenylpropanoids which have been shown to reduce chemotactic-stimulated cell migration [12, 48].

Central and peripheral nervous system inflammatory processes play an important role in the development of various persistent pathological conditions of pain [49]. Inflammatory pain is understood to be a secondary pain from that of tissue damage inflammation. Cytokines are immune system signaling molecules categorized as proinflammatory or anti-inflammatory. Studies report the involvement of proinflammatory cytokines in pathological processes of pain, including TNF-*α* and IL-1*β*, which are found at high levels in animal models of neuropathic pain. Reduction of such cytokines, both in animal models and clinical studies, has been shown to decrease painful sensation [2, 50].

In the present study, it was verified that 2-AP decreases levels of both TNF-*α* and IL-1*β* proinflammatory cytokines, suggesting that it achieves antinociception by reducing nociceptor sensitization as promoted by these inflammatory mediators.

Taylor et al. [51] demonstrated that reactive oxygen species are extremely important in the regulation of inflammation, participating in the release of arachidonic acid and subsequent formation of prostaglandins. Substances capable of sequestering free radicals inhibit all of these effects. Taking this into account, studies involving *Ginkgo biloba* extract have demonstrated its effectiveness in reducing inflammation by acting as a free radical scavenger against reactive oxygen species involved in inflammatory processes [52].

Oxidative stress occurs as a result of deregulation of the antioxidant/oxidant balance, favoring increased production of reactive oxygen or nitrogen species and leading to inflammation-related processes [53]. The antioxidant activity of 2-AP was determined through total antioxidant capacity, DPPH sequestering activity, hydroxyl radical sequestering activity, and superoxide sequestering activity tests.

The total antioxidant capacity (TAC) test determines the amount of free radicals sequestered by a sample and allows evaluation of the antioxidant capacities of natural compounds [54]. A low TAC can be indicative of oxidative stress or increased susceptibility to oxidative damage [55]. 2-Allylphenol presented a high total antioxidant capacity (TAC), justifying the use differing methodologies to determine its antioxidant activity.

The DPPH test is widely accepted as a tool for assessing antiradical activity of antioxidants. Due to the harmful effects of free radicals on biological membranes, this is extremely important [56]. However, ABTS radical scavenging assay could also be an option when it comes to samples containing hydrophilic, lipophilic, and highly pigmented antioxidant compounds [57]. In the present study, the DPPH radical sequestering activity occurred at all concentrations tested, reaching a maximum of 34.3% at 250 *μ*g mL-1, which is higher than that presented by cardanol, and which in the same concentration was also able to sequester 15.3% of the DPPH radical [58]. Oliveira et al. [48] has reported that cardanol (presenting an inhibitory concentration of 50% (IC_50_) at 3.22 *μ*g/mL) was very active in the DPPH test and highlighted its antioxidant potential as compared to the other liquid constituents tested in cashew nut shells. Though 2-AP demonstrated DPPH radical sequestering activity, it was much more discreet within its parameters of comparison.

The hydroxyl radical inhibition test was performed using the Fenton system which generates reaction between ferrous iron and hydrogen peroxide [59]. The hydroxyl radical is one of the most reactive and possesses the capacity to destroy almost all of the molecules of the cell [9].

2-AP sequestrated hydroxyl radicals in a concentration-dependent manner at the final three concentrations tested, reaching a maximum of 6.15%. This feature suggests that phenylpropanoids might be used in skin wounds, reducing OH radicals generated by UV rays [52]. The results obtained in this test indicated little capacity to sequester hydroxyl radicals, and 2-AP did not present satisfactory activity.

Superoxide radicals are produced normally in cells and exert a catalytic function towards formation of several other species of radicals. Due to the direct influence of the radical superoxide in various diseases, inhibition becomes important [60]. 2-AP presented an expressive superoxide radical sequestration percentage at all concentrations tested, obtaining a maximum value of 96.73%, at 250 *μ*g/mL. A similar study involving *Ginkgo biloba* essential oil observed inhibition of this superoxide radical by 72.24% [9].

The superoxide anion is a ROS released by macrophages and neutrophils and is responsible for activating and sensitizing nociceptive neurons, amplifying the experience of pain [4]. It was in the superoxide radical sequestration test that 2-AP presented the best performance, reaching high inhibition percentages at all concentrations tested. The data become relevant when examining antioxidant potential overall, since the superoxide radical for being able to generate other types of free radicals and oxidizing agents hides a multiplicative effect [61]. Sequestration of this ROS corroborates the results observed more specifically in the previous tests and is potentially linked to the antinociceptive effect of 2-AP.

## 5. Conclusion

**Figure 13:**
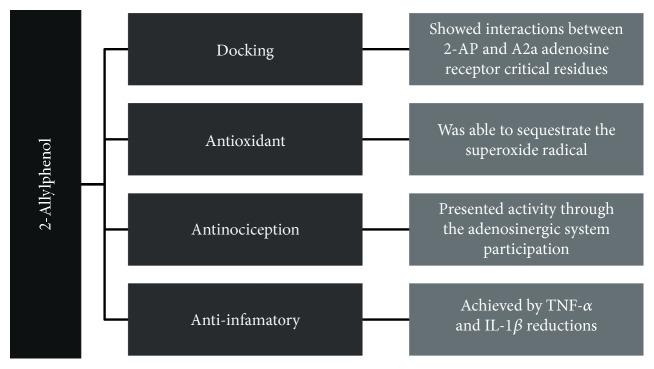
The diagram depicts all the tests performed with 2-AP in this study, emphasizing those that presented positive results.

2-AP presents antinociceptive activity via adenosinergic system participation. The anti-inflammatory properties of 2-AP involve TNF-*α* and IL-1*β* reductions, being relevant data that corroborate its antinociceptive effect. 2-AP was able to sequestrate the superoxide radical, demonstrating its antioxidant activity. Docking, interactions between 2-AP and critical residues present in the active site of the A2a adenosine receptor were identified, suggesting agonistic action for this receptor subtype. Given our results, the clinical potential of 2-allyphenol to treat pain and inflammation is evident (Figure 13).

## Figures and Tables

**Table 1:** DPPH radical scavenging ability of 2-AP (0.25, 0.5, 1, 2, and 5 mg/mL). The inhibition percentage of the DPPH radical was calculated by comparing the test results with those of the control (not treated with 2-AP) and the following equation: scavenging activity (%) = (1 − *A*1/*A*0) × 100 where *A*0 is the control absorbance and *A*1 is sample absorbances. Statistical analysis: one-way ANOVA followed by the Dunnett test.

2-Allylphenol (mg/mL)	DPPH radical scavenging (%)
0.25	34.3 ± 8.7
0.5	19.65 ± 1.48
1	18 ± 3.57
2	17.5 ± 0.35
5	18.7 ± 0.60

**Table 2:** 2-AP (0.25, 0.5, 1, 1.5, and 2 mg/mL) sequestering activity against the hydroxyl radical. Gallic acid (0.5 mg/mL) was used for positive control. The results were expressed as the percentage of hydroxyl radical-scavenging activity (%) = ([A_control_ − A_sample_]/[A_control_ − A_blank_]) × 100, where A_control_ is the control tube absorbance, A_sample_ is the sample tube absorbance, and A_blank_ is the blank tube absorbance. Statistical analysis: one-way ANOVA followed by the Dunnett test. ^a,b,c^ letters relate significant differences (*p* < 0.0001).

2-Allylphenol (mg/mL)	OH^−^ scavenging activity (%)
0.5	-0.26 ± 0.02^a^
1	1.74 ± 0.03^b^
1.5	4.01 ± 0.39^a,b^
2	6.15 ± 0.09^a,b^
Gallic acid (5 mg/mL)	97.52 ± 3.25^c^

**Table 3:** Superoxide radical sequestering activity of 2-AP (0.1, 0.25, and 0.5 mg/mL). Gallic acid (0.5 mg/mL) was used for positive control. The results were expressed as the percentage of superoxide radical-scavenging activity (%) = ([A_control_ − A_sample_]/[A_control_ − A_blank_]) × 100, where A_control_ is the control tube absorbance, A_sample_ is the sample tube absorbance, and A_blank_ is the blank tube absorbance. Statistical analysis: one-way ANOVA followed by the Dunnett test.

2-Allylphenol (mg/mL)	O_2_^−^ scavenging activity (%)
0.1	88.40 ± 4.24
0.25	96.73 ± 0.19
0.5	96.08 ± 1.11
Gallic acid (5 mg/mL)	87.22 ± 3.05

## Data Availability

The experimental, biochemical, and docking data used to support the findings of this study are included within the article. However, additional information from this study may be obtained upon request to the corresponding author (reinaldoan@uol.com.br; reinaldo@ltf.ufpb.br). We ensured that journal has the rights necessary for the proper administration of electronic rights and online dissemination of the article entitled: “2-Allylphenol Reduces IL-1*β* and TNF-*α*, Promoting Antinociception through Adenosinergic, Anti-Inflammatory, and Antioxidant Mechanisms.”
